# Targeting Host Dependency Factors: A Paradigm Shift in Antiviral Strategy Against RNA Viruses

**DOI:** 10.3390/ijms27010147

**Published:** 2025-12-23

**Authors:** Junru Yang, Ying Qu, Zhixiang Yuan, Yufei Lun, Jingyu Kuang, Tong Shao, Yanhua Qi, Yingying Li, Lvyun Zhu

**Affiliations:** College of Science, National University of Defense Technology, Changsha 410073, Chinalyf166809455612024@163.com (Y.L.);

**Keywords:** RNA virus, virus–host interaction, host dependency factor, antiviral technologies

## Abstract

RNA viruses, such as SARS-CoV-2 and influenza, pose a persistent threat to global public health. Their high mutation rates undermine the effectiveness of conventional direct-acting antivirals (DAAs) and facilitate drug resistance. As obligate intracellular parasites, RNA viruses rely extensively on host cellular machinery and metabolic pathways throughout their life cycle. This dependency has prompted a strategic shift in antiviral research—from targeting the mutable virus to targeting relatively conserved host dependency factors (HDFs). In this review, we systematically analyze how RNA viruses exploit HDFs at each stage of infection: utilizing host receptors for entry; remodeling endomembrane systems to establish replication organelles; hijacking transcriptional, translational, and metabolic systems for genome replication and protein synthesis; and co-opting trafficking and budding machinery for assembly and egress. By comparing strategies across diverse RNA viruses, we highlight the broad-spectrum potential of HDF-targeting approaches, which offer a higher genetic barrier to resistance, providing a rational framework for developing host-targeting antiviral therapies.

## 1. Introduction

**Figure 1:**
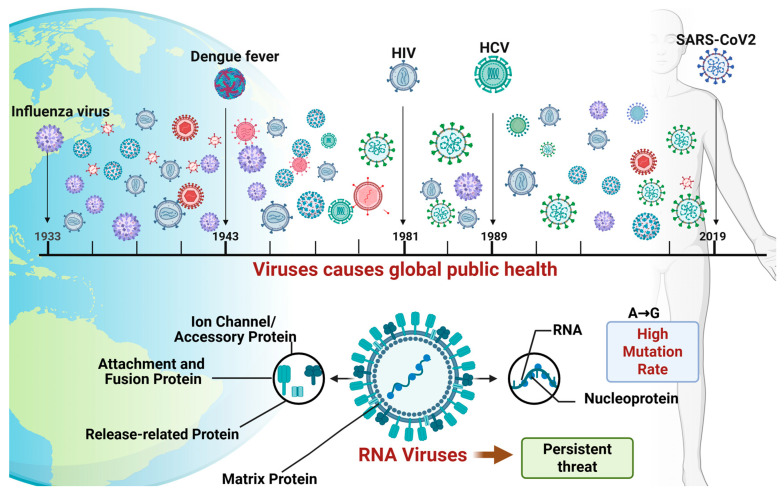
RNA Viruses: A Persistent Threat to Global Public Health Driven by High Mutability (Created in BioRender. gh, T. (2025) https://BioRender.com/qd8axoz) (accessed on 30 October 2025).

Viruses, the most streamlined biological entities in nature, are fundamentally composed of a core of genetic material (DNA or RNA) enclosed within a protein coat, or capsid. Among a myriad of viruses, RNA viruses present a persistent and formidable challenge to global public health due to their propensity for rapid mutation. This has been particularly evident in recent years, exemplified by severe acute respiratory syndrome coronavirus 2 (SARS-CoV-2) [1], which precipitated an unprecedented global health crisis; influenza virus (IV) [2], an ongoing threat responsible for millions of severe cases; and human immunodeficiency virus (HIV) [3], which has remained a global crisis for decades due to its high mortality rate and the challenges associated with its eradication. In the biological processes of virus replication, evolution, and transmission, viral nucleic acids randomly undergo mutations on one hand, and on the other hand can exchange genome fragments with other viruses through recombination mechanisms, continuously forming new viruses that are significantly different from their parental strains. These examples underscore that RNA viruses, irrespective of their genomic strategies, constitute a central threat to global public health (Figure 1).

Traditional antiviral strategies, developed from the understanding of this viral life cycle, are predicated on the direct intervention against the virus’s own components during these critical stages. These approaches primarily target the virus itself by designing small-molecule drugs or antibodies to inhibit its functions. For example, the propensity for rapid mutation in viruses such as SARS-CoV-2, influenza A virus (IAV), and HIV stems from a lack of proofreading capability during genome replication [1,4,5,6,7]. In coronaviruses and IVs, this is a characteristic of their RNA-dependent RNA polymerases (RdRp) [7], whereas for HIV, its hallmark reverse transcriptase exhibits a high error rate when converting RNA into DNA [8]. Therefore, traditional antiviral strategies can be classified into two categories. The first involves neutralizing antibodies that block viral entry, for instance, by targeting the Spike (S) protein of SARS-CoV-2 or the Hemagglutinin (HA) of IV, a concept that also underpins mainstream vaccine design [9,10,11]. The second category focuses on the development of small-molecule inhibitors that target key viral enzymes essential for replication [12,13,14], such as the RNA-dependent RNA polymerase (RdRp), proteins from the NSP family, or the 3CL protease [15,16]. This line of research has yielded drugs like Paxlovid, Molnupiravir, and Favipiravir [17,18,19]. Additionally, inhibitors targeting specific viral functions, such as neuraminidase (NA) inhibitors and M2 ion channel blockers for the IV, have also served as important therapeutic options [20,21]. However, all these strategies targeting the virus directly, known as direct-acting antivirals (DAAs), are fundamentally challenged by a core feature of RNA viruses: their high mutation rate. For example, key surface proteins, such as the S protein of SARS-CoV-2, the HA of IV, and the envelope glycoprotein (gp120) of HIV, are frequent mutational hotspots [7,22,23]. This rapid genetic variation enables the virus to continuously select for mutant strains that evade the effects of drugs and vaccines, leading to the development of drug resistance and immune escape. This, in turn, diminishes or completely negates the efficacy of the aforementioned therapies, posing a persistent threat to public health [24,25,26,27].

The intrinsic limitations of the above “virus-targeting” paradigm have catalyzed a shift in research toward targeting host dependency factors (HDFs), which are highly conserved across species. HDFs are cellular proteins that viruses exploit to complete their life cycle, serving as potential antiviral drug targets. This transition from targeting the mutable virus to the relatively stable host is grounded in the fundamental nature of viruses as “minimalist parasites”. Lacking independent metabolic and protein synthesis machinery, every stage of their life cycle necessitates the hijacking of host cellular machinery. This fundamental dependency presents a novel therapeutic avenue: targeting HDFs. A host-oriented strategy offers two primary advantages: first, the potential for broad-spectrum efficacy, as divergent viruses may rely on common host pathways; and second, a higher barrier to resistance, as it is substantially more difficult for a virus to evolve independence from essential host functions through simple mutations. The feasibility of this host-centric approach is not merely theoretical but has been validated by a series of successful examples, from established drugs to compounds in clinical development. The licensed broad-spectrum antiviral drug Ribavirin, for instance, effectively suppresses viral proliferation by interfering with host nucleotide metabolism [28]. Similarly, 2-Deoxy-D-glucose (2-DG), a glycolysis inhibitor currently in clinical trials, has been shown to potently inhibit viral replication by disrupting cellular energy pathways [29]. At the viral entry stage, the approved anti-HIV drug Maraviroc functions by antagonizing the host co-receptor CCR5, which is essential for viral entry [30]. In post-entry stages, novel drugs such as GS-CA1, currently in clinical development, inhibit HIV by disrupting the integrity of the incoming capsid core and its critical interactions with host factors [31]. The paradigm of antiviral drug development is undergoing a profound transformation, marked by a strategic shift from traditional DAAs to Host-Targeting Antivirals (HTAs) [32,33,34]. Although DAAs have historically predominated, the proportion of HTAs in emerging clinical pipelines has increased significantly, giving rise to a new frontier of combination therapies, exemplified by the “DAA + HTA” approach.

This strategic evolution from DAA to HTA is fundamentally enabled by revolutionary breakthroughs in underlying technologies, particularly innovations in genome-wide screening tools. Gene-editing technologies, such as CRISPR-Cas9, have been instrumental in systematically and precisely identifying the HDFs essential for the viral life cycle [35,36,37,38]. It is this expansive landscape of “druggable” host targets, unveiled by technological innovation, that has transformed the host-targeting strategy from a theoretical concept into a viable pathway for research and development.

In this review, we systematically dissect host dependencies throughout their entire life cycle, highlighting key HDFs and their mechanisms of hijacking. We also emphasize how cutting-edge HDF discovery tools, including CRISPR screening, for identifying the HDF landscape, are directly fueling the development of next-generation therapeutics, thereby providing the foundation for a strategic shift towards HTAs and their use in advanced combination therapies, aiming to provide a comprehensive framework for developing novel antiviral therapies.

## 2. Brief Introduction of General RNA Viruses

### 2.1. The Classification of RNA Viruses

**Figure 2:**
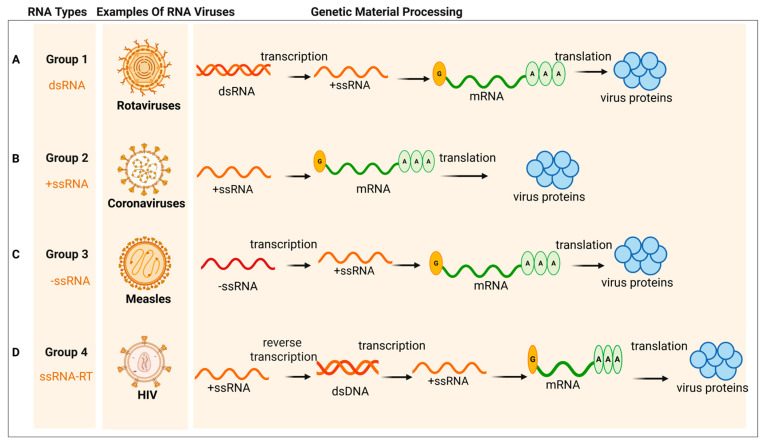
The Classification of RNA Viruses and their distinct strategies for messenger RNA (mRNA) synthesis and virus proteins. (**A**) Double-stranded RNA (dsRNA) viruses. (**B**) Positive-sense single-stranded RNA (+ssRNA) viruses. (**C**) Negative-sense single-stranded RNA (−ssRNA) viruses. (**D**) Single-stranded RNA retroviruses (ssRNA-RT). (Created in BioRender. gh, T. (2025) https://BioRender.com/885dvc9) (accessed on 30 October 2025).

**Figure 3:**
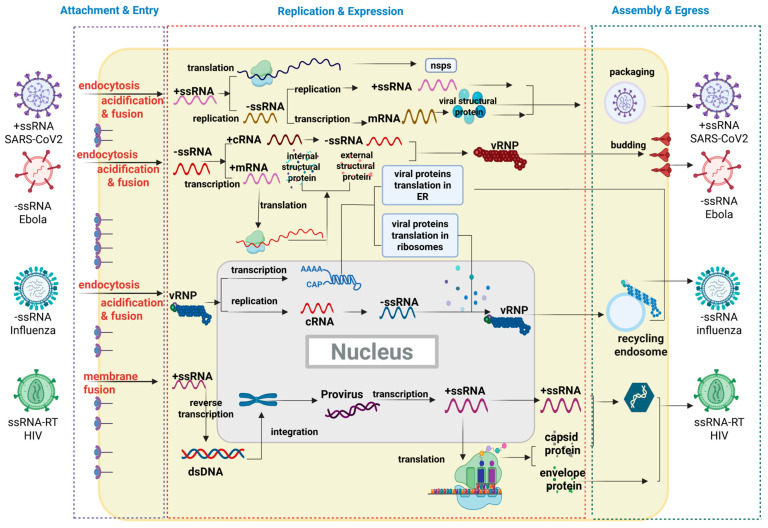
A comprehensive scheme of the viral life cycle mapping key host dependency factors to distinct stages of infection (Created in BioRender. gh, T. (2025) https://BioRender.com/lxoivpn) (accessed on 27 November 2025).

According to the Baltimore classification system, RNA viruses can be divided into four major groups based on their distinct strategies for messenger RNA (mRNA) synthesis [39] (Figure 2 and Figure 3). As shown in Figure 2A, for double-stranded RNA (dsRNA) viruses, the virion-packaged RNA-dependent RNA polymerase (RdRp) transcribes the negative-sense strand of the dsRNA into +ssRNA within the viral core, which is then exported to the cytoplasm. In contrast, the genomic RNA of positive-sense single-stranded RNA (+ssRNA) viruses (Figure 2B) is itself mRNA. It can be immediately translated directly by the host ribosomes, producing viral proteins (including RdRp). This virus-encoded RdRp is then essential for the subsequent replication step: using the +ssRNA as a template to synthesize a complementary negative-sense single-stranded RNA (−ssRNA). This −ssRNA strand is then used as a template to synthesize large amounts of progeny +ssRNA. These progeny +ssRNA molecules can, in turn, be translated into more proteins or serve as the genome for new viral particles. Negative-sense single-stranded RNA (−ssRNA) viruses (Figure 2C) use the −ssRNA as a template to synthesize complementary +ssRNA. These +ssRNA molecules are the mRNA, which are translated into various viral proteins. Simultaneously, the virus also synthesizes full-length +ssRNA to use as a template for the mass replication of progeny −ssRNA genomes. The +ssRNA serves as messenger RNA (mRNA) for translation into various viral proteins, while the virus also synthesizes full-length +ssRNA molecules to serve as templates for the mass production of progeny −ssRNA genomes. As shown in Figure 2D, single-stranded RNA retroviruses (ssRNA-RT) employ a unique reverse transcription step, converting their RNA genome into a DNA intermediate that integrates into the host chromosome, from which mRNA is subsequently transcribed by the host’s RNA polymerase II. Given that dsRNA viruses are relatively less common in terms of species diversity, the following discussion will primarily focus on +ssRNA, −ssRNA, and ssRNA-RT viruses.

### 2.2. The Life Cycle of RNA Virus

**Figure 4:**
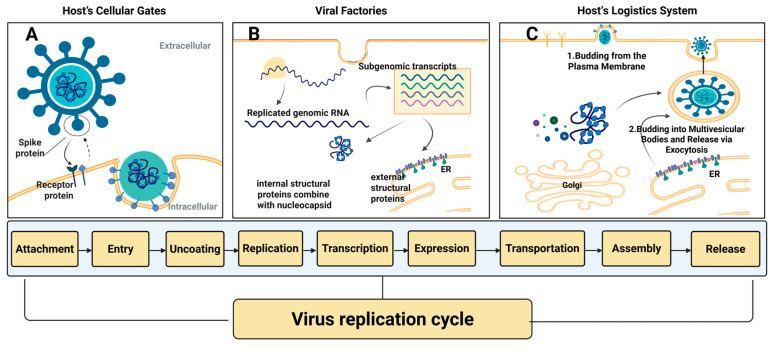
Virus Replication Cycle. (**A**) Hijacking the Host’s Cellular Gates: Attachment and Entry. (**B**) The Viral “Factories”: Replication and Expression. (**C**) Targeting Host Dependency Factors of the Host’s Logistics System. (Created in BioRender. gh, T. (2025) https://BioRender.com/f95a0w0) (accessed on 30 October 2025).

Despite these vast structural and strategic differences, as obligate intracellular parasites, all viruses are entirely dependent on the host cell’s ability to complete their RNA replication cycle and expression (Figure 4). This process is generally delineated into several critical stages: (i) attachment and entry (Figure 4A); (ii) replication and expression (Figure 4B); (iii) assembly and egress (Figure 4C). Underpinning this complex series of events, viruses do not operate in isolation; instead, they drive their life cycle by manipulating a multitude of Host Dependency Factors (HDFs). Therefore, a thorough understanding of how viruses hijack HDFs at each stage is a prerequisite for the systematic discovery of new therapeutic targets. Accordingly, next part will follow the virus’s life trajectory from entry and replication to assembly and egress, to dissect these critical dependencies and elucidate corresponding potential novel host dependency factors (HDFs) viable for antiviral targeting.

## 3. Breaching the Invasion Gateway

### 3.1. Hijacking the Host’s Cellular Gates: Attachment and Entry

The initiation of viral infection commences with binding to and entry into the host cell via specific surface receptors. The virus utilizes glycoproteins on its envelope to engage with host receptor molecules, a process analogous to a “key” fitting into a “lock”. As shown in Table 1, the receptors through which viruses enter host cells are not the same and are specific to the virus. For instance, Ebola virus (EBOV; −ssRNA) requires host cholesterol to enhance its binding to Niemann-Pick C1 protein (NPC1) within host endosomes for entry [40]. Hepatitis C virus (HCV; +ssRNA) depends on its envelope proteins interacting with four key host receptors, namely SR-BI, CD81, Claudin-1, and Occludin, culminating in entry via clathrin-mediated endocytosis [41]. SARS-CoV-2 (+ssRNA) primarily enters cells through a pathway mediated by angiotensin-converting enzyme 2 (ACE2) receptor [42,43]. For IV (−ssRNA), after its HA protein binds to conventional sialic acid receptors for initial attachment, it must also directly interact with the host metabotropic glutamate receptor 2 (mGluR2) to mediate endocytosis [44]. Chikungunya Virus (CHIKV; +ssRNA) utilizes its E1/E2 glycoproteins to bind with multiple receptors, such as MXRA8 and Prohibitin-1, entering the cell via the endocytic pathway [45]. E protein of Zika Virus (ZIKV; +ssRNA), in turn, binds to receptors like AXL, Tyro3, and TIM-1 to achieve tropism for neural progenitor cells and placental cells [46]. For non-enveloped viruses, such as Enterovirus A71 (EV-A71; +ssRNA) [47] and Coxsackievirus B3 (CVB3; +ssRNA) [48], they rely on their VP1 capsid protein to recognize receptors like SCARB2 and CAR, respectively, inducing conformational changes and initiating the endocytic process. Finally, the highly pathogenic Nipah Virus (NiV; −ssRNA) uses its G and F glycoproteins to bind with the host cell surface receptors Ephrin-B2 and Ephrin-B3, directly mediating membrane fusion on the cell surface to complete entry [49].

This “key-in-lock” receptor binding is merely the first step of viral entry. For the ultimate delivery of its genome, the virus must also exploit the host’s protease systems, signal pathways or the endosomal environment to trigger the subsequent steps of membrane fusion and genome release. The diverse entry mechanisms of coronaviruses, which allow them to adapt to different host cell types, serve as a prime example of how viruses hijack host factors for entry. These strategies can be broadly categorized into two pathways: cell surface fusion and the endocytic route. The former is dependent on the cleavage and activation of the viral S protein by cell surface proteases, such as Transmembrane Serine Protease 2 (TMPRSS2) [50,51]. The latter relies on the host AP2-associated kinase 1 (AAK1) to regulate clathrin-mediated endocytosis. Following successful viral endocytosis, it utilizes the low pH environment of endosomal acidification to activate cathepsins for cleavage [52,53]. Beyond the specificity of entry pathways, the regulatory role of the host’s internal microenvironment is critical. The acidic microenvironment is not only a prerequisite for the activation of cathepsins but also acts as a key molecular switch in itself. It triggers dramatic conformational changes in viral glycoproteins, thereby initiating the molecular program required for executing membrane fusion and genome release.

Furthermore, viral invasion involves the precise manipulation of host signaling pathways. For instance, after the HA protein binds to the metabotropic mGluR2, it rapidly activates the host’s p38 MAPK signaling pathway. This signaling event is far from being merely a cellular stress response; rather, it constitutes an essential step in the viral entry program—the activated p38 signal acts as a critical trigger that induces membrane invagination, thereby proactively initiating the endocytosis of viral particles [54]. Furthermore, other viruses, such as Respiratory Syncytial Virus (RSV), can initiate this pathway through the recognition of viral surface proteins by Toll-like receptor 4 (TLR4) on the cell membrane. This signaling is transduced via the adaptor protein MyD88 to downstream components [54]. Since many viruses across different families require its activation, inhibiting p38 holds promise for developing broad-spectrum antiviral drugs effective against multiple viruses. With intervention strategies spanning multiple tiers of the signaling cascade, the kinase itself serves as a central hub, and its specific inhibitors effectively block viral internalization. Downstream p38-mediated phosphorylation of endocytic regulatory proteins such as EEA1 and Rabenosyn-5 is critical for promoting viral entry [55,56], while phosphorylation of the transcription factor ATF-2 serves as a key readout of pathway activation [57,58]. This multi-tiered targeting strategy—focused on host cellular machinery rather than viral components—offers a novel and broad-spectrum approach to antiviral intervention [54].

During the subsequent intracellular transport and uncoating stage, epigenetic enzymes play an unexpected mechanical role. The host protein HDAC6 specifically recognizes and binds to the unanchored ubiquitin chains on the IAV capsid, acting as a molecular bridge that connects the virus to dynein motors transporting along microtubules toward the nucleus. Meanwhile, the viral capsid is subjected to traction from opposing actin-based motors. This bidirectional molecular motor “tug-of-war” generates substantial shear forces on the capsid, physically tearing it open and releasing the viral ribonucleoprotein (vRNP), thereby completing the final step of viral invasion [59,60]. However, the strategies and mechanisms by which different viruses utilize HDAC6 vary significantly. The strategy employed by ZIKV (Zika Virus) is more complex: while studies show that its non-structural protein NS5 promotes microtubule (MT) acetylation, thus altering the cytoskeleton, HDAC6 acts as an anti-ZIKV factor by promoting the autophagic degradation of NS5; yet, similar to IAV, other research indicates that ZIKV infection is also influenced by the HDAC6 ubiquitin-binding zinc finger (ZnF) domain, potentially involving uncoating, suggesting its strategy encompasses cytoskeletal alteration and evasion of HDAC6-mediated autophagic degradation [61]. In terms of immune regulation, other viruses (such as Vesicular Stomatitis Virus, VSV) exploit the deacetylase activity of HDAC6 to deacetylate the STING protein (at the K338 site), thereby negatively regulating the cGAS-STING signaling pathway and suppressing the production of Type I interferons (IFN-I), which represents a classic strategy for immune evasion to promote viral replication [62]. For HIV (Human Immunodeficiency Virus), studies indicate that inhibiting HDAC6 (e.g., via knockdown or inhibitors) can enhance HIV-1 infection in primary lymphocytes. This suggests that HDAC6 may function as an anti-HIV factor in certain cells or stages, and its mechanism may relate to influencing host immune responses or specific viral life cycle steps (such as integration or reverse transcription), though the detailed mechanism lacks a direct correlation with the aforementioned physical uncoating and immune regulation strategies [63]. In conclusion, HDAC6 plays diverse and critical roles across different stages and mechanisms of viral infection, rendering it a promising antiviral target.

As established, the presence of specific receptors serves as the fundamental prerequisite for determining viral tropism. However, successful viral entry is a process far more intricate than a singular “key-in-lock” recognition. It is contingent upon a “permissive cellular microenvironment” governed by a multitude of host factors, from which the virus selectively exploits key elements according to its unique entry strategy. These critical determinants include the host’s protease expression profile, the kinetics of endosomal acidification, and the lipid composition of the cell membrane. Notably, emerging evidence underscores that this permissive environment is further orchestrated by the host’s epigenetic machinery and kinase signaling networks, which actively regulate the mechanical and signaling checkpoints essential for viral internalization. Consequently, this array of host factors, spanning from receptor presentation on the cell surface to the complex intracellular landscape of enzymatic activity, signaling cascades, pH gradients, and lipid dynamics, collectively constitutes the critical dependency network that ultimately dictates the success of viral invasion.

### 3.2. Targeting HDFs in Viral Entry

Host receptors, as the “first point of contact” in virus–host interactions, as concluded in Table 2, have emerged as the most direct and tractable therapeutic targets, leading to significant early progress in antiviral drug development. Strategies in this domain primarily operate on three levels: direct inhibition of the receptor’s enzymatic active site with small molecules; blockade of virus-receptor binding using high-affinity ligands; or downregulation of receptor synthesis and surface presentation through the modulation of gene expression [64,65,66,67]. In stark contrast, drug development targeting other critical nodes of viral entry, such as the host protease system, endosomal acidification, and lipid metabolism networks, epigenetic machinery and kinase signaling networks, has comparatively lagged. The fundamental reason for this disparity is not the inefficacy of these targets, but rather their “indispensability” to the host cell’s basic physiology, which introduces an inherent risk of cytotoxicity. This challenge is clearly illustrated by specific drug examples. For instance, chloroquine, which targets endosomal acidification, exerts its antiviral effect by disrupting the pH gradient essential for the endocytic pathway. However, this systemic interference with a fundamental cellular transport system also accounts for its off-target toxicity, thereby limiting its therapeutic application. Similarly, statins, which target lipid metabolism, exhibit antiviral potential by inhibiting the cholesterol synthesis pathway, yet their side effects in clinical use highlight the risks associated with long-term intervention in this core metabolic network. Furthermore, all clinically developed HDAC6 inhibitors target its catalytic domains and fail to block the ZnF-UBP-mediated physical uncoating process [68]. Compounding this issue, HDAC6 itself serves as a critical activator of host innate immunity, such as in the RIG-I pathway, inhibiting its enzymatic activity severely impairs interferon responses. This creates an “immunological paradox”, whereby blocking viral entry may simultaneously exacerbate infection [69]. The host protease network presents a similar dilemma; its members play pleiotropic roles in cellular signaling, protein homeostasis, and apoptosis, making the development of inhibitors that are both highly specific and safe a formidable task [70,71].

Therefore, the core impediment to drug development in this area is not a lack of drug potency, but rather the difficulty of achieving effective viral inhibition within a narrow therapeutic window without inflicting unacceptable collateral damage on the host. This challenge, however, also illuminates the path forward: a more precise identification and intervention against those pathway nodes that are “preferentially hijacked” by viruses. Such a focused approach holds the potential to create the next generation of antiviral therapies that combine broad-spectrum activity, a high barrier to resistance, and host safety.

## 4. Decoding the Viral “Factories”

### 4.1. The Viral “Factories”: Replication and Expression

After the viral genome enters the host cell, its primary task is to “hijack” and reshape the host’s endomembrane system to establish an efficient and concealed “replication factory”, known as viral replication organelles (VROs) [85]. The construction of these compartmentalized structures is underpinned by the biophysical principle of Liquid–Liquid Phase Separation (LLPS), which is the result of macromolecular self-assembly driven primarily by multivalent interactions between proteins and nucleic acids [86,87,88,89]. Many viral and host proteins leverage Intrinsically Disordered Regions (IDRs) to facilitate these weak and transient interactions, which are crucial for phase separation to occur. These IDRs lack a fixed tertiary structure, providing the necessary flexibility and multivalency. This dynamic compartmentalization carries significant functional implications: the resulting condensates are considered “reaction crucibles”, vastly concentrating viral enzymes and essential cofactors, thereby enhancing the spatial and temporal specificity and efficiency of various stages of the viral life cycle. By locally creating high-concentration environments, the virus can optimize its complex molecular processes, avoiding the inefficiency of reactions in the diluted cytoplasmic environment [90,91].

Many +ssRNA viruses are particularly adept at remodeling the host cell’s endomembrane system to construct their VROs. These cytoplasmically formed VROs can be categorized into different types: “Spherules” induced by flaviviruses (e.g., dengue virus, DENV (+ssRNA); HCV) on the endoplasmic reticulum (ER) membrane, a process mediated by their non-structural (NS) proteins such as NS1 and NS4A/4B [92]; VROs formed by coronaviruses through the interaction of non-structural proteins nsp3 and nsp4. The initiation of this process depends on TMEM41B—the first key HDF—which mediates the binding of nsp3 to nsp4, thereby triggering the “zippering” of ER membranes. A second key HDF, VMP1, is then required to ultimately convert these membranes into closed, spherical double-membrane vesicles (DMVs) [93]. At the center of the pore in the final DMV, there is a “ring” composed of positively charged arginine residues, which enables the newly synthesized viral RNA to be exported from the interior of the DMV to the cytoplasm [94]. As for −ssRNA viruses, filoviruses (e.g., EBOV) undergo massive accumulation of their nucleoprotein (NP) in the cytoplasm, which spontaneously condenses to form membrane-less “viral inclusion bodies”. The formation of these inclusion bodies is a canonical example of LLPS, where the NP often possessing key IDRs and multivalent binding domains, drives the phase separation process to recruit and organize the entire viral RNA synthesis machinery [95]. The NP of EBOV directly binds to the polymerase cofactor VP35 via its central domain, initiating internal RNA synthesis. The host kinase system exerts dual regulation on EBOV: it not only acts as a functional switch for viral transcription and replication by phosphorylating VP30 but also modulates VP35’s immune antagonistic activity through phosphorylation [96,97]. Additionally, NP recruits carbamoyl-phosphate synthetase 2, aspartate transcarbamylase, and dihydroorotase (CAD) to inclusion bodies via its glutamine (GLN)-containing domain, thereby providing pyrimidines for EBOV genome replication and transcription [98].

The LLPS mechanism not only dramatically enhances viral genome replication efficiency by forming concentrated replication crucibles, but also serves as a core organizational principle for both viral replication and immune evasion, establishing it as a critical target for HTAs [99]. In the early stages of viral replication, LLPS mechanism emerges as a key strategy for the virus to facilitate immune evasion. The host defense system utilizes core HDFs—G3BP1/2—to mediate LLPS, forming Stress Granules (SGs) that restrict viral replication by sequestering the translation machinery and antiviral factors, making SGs a vital component of the innate antiviral response. To evade this, viruses have evolved highly specialized strategies to manipulate this core mechanism, making G3BP1/2 a crucial node in the virus–host interactome. On one hand, certain viruses (such as SARS-CoV-2) employ a “Competitive Disruption Mode”: their proteins (like the N protein) engage in co-LLPS with G3BP1/2, physically occupying the G3BP interaction sites. This leads to the disassembly of SGs, thereby effectively inhibiting the innate immune response. Concurrently, viruses directly interfere with host signaling hubs: for instance, SARS-CoV-2 N protein, through competitive LLPS, displaces MAVS-driven droplet formation and inhibits MAVS polymerization, directly disrupting the MAVS signaling pathway [100,101]. On the other hand, other viruses (such as CHIKV and Norovirus) actively co-opt G3BP1/2 for their own life cycles. For example, Old World CHIKV utilizes the FGxF motif on its non-structural protein nsP3 to bind with G3BP. This binding mimics the action of the host proteins Caprin-1/USP10, effectively recruiting G3BP1 to the viral replication complex (VRC) assembly sites. Murine and human Noroviruses similarly rely on G3BP1 for early replication and translation, where the silencing of G3BP1 impairs viral replication [102]. In summary, the LLPS mechanism, serving as a core organizational principle for both viral replication and immune evasion, represents a critical target for HTAs [103].

New drugs are currently being developed, aiming to directly interfere with the multivalent interactions or IDR function that drive LLPS [101]. For instance, various small molecules and peptide inhibitors are being investigated to block receptor interactions and the LLPS process [104]. Given that the formation of LLPS condensates is highly dependent on the host cell’s internal environment from a physicochemical perspective (e.g., ionic strength, pH) and post-translational modifications (e.g., host kinase-mediated phosphorylation), this reveals a promising new antiviral strategy: using pharmacological approaches to regulate these host pathways [105]. For instance, interfering with the multivalent interactions or the ionic environment that drives LLPS can destabilize viral condensates. This regulation targeting the internal environment and molecular interactions can indirectly interfere with the stability and function of replication organelles (ROs) or directly target RO formation, ultimately inhibiting viral replication [106,107,108].

The construction of these large-scale membrane structures involves not only remodeling the morphology of organelles but also, more fundamentally, profound reprogramming of the host’s metabolic network by the virus [109,110,111]. To meet the requirements for volume, curvature, and fluidity of RO vesicle membranes, +ssRNA viruses hijack numerous host pathways involved in lipid synthesis, modification, and transport [112,113]. In terms of viral remodeling of the host endomembrane system, +ssRNA viruses induce membrane bending and invagination by hijacking host curvature-inducing proteins such as ESCRT (Endosomal Sorting Complex Required for Transport) and SNX-BAR (Sorting Nexin-Bin/Amphiphysin/Rvs), forming the “spherule” structures required for their replication. They also rely on the actin network to provide structural support. For viruses that need to enter the nucleus, they depend on key nuclear import factors such as host dyneins and karyopherins to actively and orderly complete processes including stabilizing themselves, regulating uncoating, directional transport, docking at nuclear pores, and finally entering the nucleus [114]. In the context of lipid metabolism, viruses do not merely hijack host enzymes responsible for the production and transport of specific lipids; instead, they hijack the entire lipid metabolic network to create an environment conducive to their own replication. Cholesterol metabolism supports viral entry and replication: SARS-CoV-2 uses proliferated lipid droplets as a “logistical raw material reservoir” to supply lipid precursors for membrane synthesis of VROs [115,116]. Imipramine and ceftanorine are FDA-approved drugs that target cholesterol metabolic pathways [117]; Furthermore, SARS-CoV-2 uses different proteins to regulate host cell processes to promote infection. ORF9b is localized in the mitochondria and binds to Tom70, suppressing the type I interferon pathway to achieve immune evasion, while ORF7a induces autophagosome accumulation and blocks their fusion with lysosomes, creating a favorable niche for viral replication [118,119]; DENV adopts a more direct strategy: it induces autophagy to degrade and consume lipid droplets, using them as an “immediate fuel source” for energy production [120]. The resulting breakdown products are subsequently channeled into β-oxidation, yielding high-energy ATP and essential lipid precursors, thereby substantially accelerating the dual requirements of energy supply and membrane biogenesis necessary for viral replication. Crucially, DENV infection necessitates the central metabolic regulator AMPK for the initiation of lipophagy [121,122]. This indicates that targeting the lipid synthesis or transport pathways hijacked by viruses is an effective antiviral strategy. Pharmacological suppression of the host lipid metabolic network, including triglycerides, sterols, and multiple phospholipids, or targeted intervention in the autophagy-lipid mobilization axis, can effectively sever the energy and membrane material supply to viral factories, thereby blocking viral replication. This strategy provides a key direction for developing broad-spectrum antiviral agents with a high resistance barrier [115,123,124].

While establishing their replication factories, viruses must disrupt or hijack the host’s immune alarm system. Mitochondria-associated membranes (MAMs), specialized contact sites between the endoplasmic reticulum and mitochondria, serve as physical scaffolds that drive innate immune signaling [125]. Because MAMs concentrate key signaling adaptors such as MAVS, they represent a critical defense hub that viruses often target for immune evasion [125]. At this MAM-MAVS immune synapse, MAVS (Mitochondrial Antiviral-Signaling proteins) activates IRF3 and promotes interferon (IFN) production. HCV exploits the host’s reliance on this platform: its NS3/4A protease localizes precisely to MAMs and cleaves the MAM-anchored MAVS pool, thereby blocking RIG-I signaling at its origin. This direct cleavage represents a viral strategy to dismantle the host’s primary immune signaling platform, highlighting the importance of MAM localization in targeting MAVS for HTA strategies [126]. Furthermore, within MAMs, MAVS interacts with GFPT2 (the rate-limiting enzyme of the hexosamine biosynthesis pathway (HBP)) to activate HBP, coupling metabolic status to sustained IFN-I responses. Notably, GFPT2 is a host metabolic enzyme upregulated during antiviral responses to enhance MAVS signaling through O-GlcNAcylation; it is not a factor that viruses depend on for replication, but rather a host defense component that viruses may indirectly suppress [127]. GFPT2 thus represents a potential host target for immunomodulation [128].

Peroxisomes constitute another unique innate immune platform, where compartmentalized MAVS (MAVS-Pex) initiates rapid, IFN-independent defense—such as activating the ISG viperin [129]. Effective viral restriction requires coordinated signaling between MAVS-Pex (for rapid containment) and mitochondrial MAVS (for delayed, sustained responses). To escape this early line of defense, ZIKV actively disrupts peroxisomal homeostasis, leading to peroxisome depletion. Studies show that PEX11B, a key factor in peroxisome biogenesis, can be enhanced to amplify MAVS-Pex signaling and suppress ZIKV replication [130]. Therefore, targeting host factors such as PEX11B to stabilize or enhance immediate immune platforms offers a promising approach for HTA.

### 4.2. Targeting HDFs in Viral Replication

The replication and integration of viruses are also subject to precise regulation by host factors. From the very beginning of infection, viruses begin to manipulate the internal environment of cells; for instance, they utilize the host’s reactive oxygen species (ROS) generation pathways to regulate the redox state, thereby creating favorable conditions for early-stage replication [131]. In the core processes of genomic replication and transcription, viruses exhibit a particularly strong reliance on host polymerases and related cofactors: IAV binds to host RNA polymerase II to implement a “cap-snatching” strategy for obtaining transcription primers, and it depends on ANP32 family proteins to support its polymerase activity [132]; in contrast, some other viruses recruit host DNA topoisomerase I (TOP1) to assist in RNA synthesis [133,134]. Beyond their dependence on polymerases, signaling pathways that act as “replication switches” are also targets for viral manipulation. Although NF-κB is typically a hallmark of antiviral immunity, RNA viruses such as IAV and SARS-CoV-2 exhibit dependence on it. For IAV, synthesis of viral cRNA (a replication intermediate) relies on NF-κB activity. Inhibiting IKK or NF-κB (e.g., using SC75741) specifically blocks viral RNA synthesis [135,136]. Similarly, for SARS-CoV-2, the NF-κB transcriptional footprint is crucial for efficient replication, which creates a paradox: the virus drives inflammation to support its own growth [137]. This critical stage of replication initiation and genome processing is subject to even deeper regulation for retroviruses, regarding their genome integration: the integration of their genomes not only requires the anchoring of “guidance proteins” such as LEDGF/p75 [138], but is also fundamentally influenced by the physical properties of the host genome (e.g., DNA flexibility and three-dimensional chromatin accessibility) [139]. Thus, these critical host factors hijacked by viruses, encompassing ROS regulatory pathways, polymerase cofactors, NF-κB signaling switches, and integration proteins such as LEDGF/p75, collectively constitute a core network essential for viral replication, thereby providing significant targets for the development of host-directed antiviral drugs (as concluded in Table 3).

Additionally, the host’s epigenetic mechanisms, including RNA/DNA methylation and histone modification, also exert precise regulation on viral gene expression [140]. During processes such as chromatin remodeling and viral protein modification, viruses hijack the host acetylation machinery. A representative example is the association between HDAC1 and the influenza viral NP protein: host HDAC1 is recruited to interact with NP and regulates its acetylation state at lysine 103 (K103). Studies have shown that HDAC1 promotes the nuclear retention of NP, and the deacetylated state of K103 is associated with enhanced viral replication [141]. After viral transcripts are translated into proteins, their correct folding, processing, and stabilization are entirely dependent on the host’s molecular machinery. This machinery includes the Sec61 translocon, the oligosaccharyltransferase (OST) complex, and factors like Sec24C, which are crucial for the capsid stability of specific viruses (e.g., HIV) [142]. Notably, during the life cycle of viruses such as flaviviruses, the dolichol-phosphate-mannose synthase (DPMS) complex and the mannosyltransferase ALG3 in the endoplasmic reticulum play dual roles: they are involved in RNA replication and are also essential for the folding and stability of viral structural glycoproteins [143]. Studies have identified broad-spectrum host factors that are commonly hijacked by different viral families, such as the involved in ribosome biogenesis, as well as the vacuolar ATPase and the conserved oligomeric Golgi (COG) complex [144].

Whether targeting host pathways, the internal cellular environment, or host factors, the profound reliance of viruses on host networks reveals a large number of potential host-directed therapeutic targets [145,146]. Furthermore, by inhibiting key nodes in certain inflammation-related pathways, these targets emerge as prime examples of a “dual mechanism” approach, possessing both anti-inflammatory and antiviral efficacy, perfectly illustrating the advantages of HTAs. However, translating these findings into clinical drugs still faces challenges. The three primary challenges currently are: the complexity of drug design targeting intrinsically disordered proteins and multivalent interactions [147], the difficulty in achieving high specificity for pathological condensates to avoid systemic toxicity, and most critically, the lack of reliable clinical pharmacodynamic biomarkers to guide dosing and validate efficacy [148]. Therefore, currently approved antiviral drugs mainly consist of inhibitors targeting key viral enzymes or interferons [149], which underscores the urgent need to further explore the druggability and safety of these shared HDFs.

**Table 3:** Therapeutic strategies targeting key host dependency factors during the viral replication and expression stageAmong the marketed drugs, there are drugs that have been repurposed.

Host Dependency Factor (HDF)	Virus Example	Clinical States	Targeting Drug/Strategy
TMEM41B [93]	Coronaviruses	/	/
VMP1 [93]	Coronaviruses	/	/
Host Kinase System [96,97]	Ebola Virus (EBOV)	/	/
CAD (Carbamoyl-Phosphate Synthetase 2) [98]	Ebola Virus (EBOV)	/	/
Host Nucleotide Metabolism	Various RNA viruses	In clinical development	Ribavirin [150]
Host Curvature-inducing Proteins [114]	Various RNA viruses	/	/
Host’s Lipid Metabolic Network	Severe Acute Respiratory Syndrome Coronavirus 2 (SARS-CoV-2)	In clinical development	Imipramine, ceftanorine [117]
RNA Polymerase II [132]	Influenza A virus (IAV)	/	/
ANP32 Family Proteins [132]	Influenza A virus (IAV)	/	/
	Various RNA viruses		
TOP1	Various RNA viruses	In clinical development	Camptothecin [151]
LEDGF/p75 [138]	Retroviruses (HIV)	/	/
Sec24C [142]	Human Immunodeficiency Virus (HIV)	/	/
DPMS Complex & ALG3 [143]	Flaviviruses	/	/
Glycolysis Pathway	Various RNA viruses	Marketed drugs	2-Deoxy-D-Glucose (2-DG) (in Phase 1/2/3 clinical trial) [29]
SBDS & SPATA5 proteins [144]	Flaviviruses & Coronaviruses	/	/
LLPS	Various RNA viruses	/	/
NF-κB	IAV & SARS-CoV-2	/	
HDAC1	IAV & SARS-CoV-2	Marketed drugs	Valproic Acid (VPA) [152]
GFPT2	HCV	/	/
PEX11B	ZIKV	/	/
LC3	SARS-CoV-2	/	/
AMPK	Various RNA viruses	/	/
G3BP1/2	SARS-CoV-2	Marketed drugs	Imatinib, Decitabine [105]

## 5. Commandeering the Host Logistics

### 5.1. Commandeering the Host’s Logistics System: Assembly and Egress

The assembly and release, the final stages of the viral life cycle, are a series of events that require precise spatiotemporal coordination. During this phase, viruses fully hijack the host’s vesicular trafficking and membrane remodeling/scission machinery.

For RNA viruses that replicate within the nucleus, the primary task is the export of their genomes to the cytoplasm. This process predominantly relies on the Nuclear Pore Complex (NPC) pathway to traverse the nuclear envelope. HIV-1, for example, utilizes its Rev protein as a “molecular adaptor” to indirectly link the naked RNA genome to the CRM1 export system, thereby facilitating active export. IAV employs an even more elaborate strategy. It not only utilizes the CRM1 pathway, but the “cargo” it transports is no longer a simple RNA molecule but rather a large, packaged viral ribonucleoprotein (vRNP) complex. Concurrently, its newly synthesized viral mRNAs are exported through the NPC via diverse pathways, including the NXF1-mediated route, demonstrating a differential transport strategy for distinct types of cargo [153,154,155].

Regardless of whether genome replication occurs in the nucleus or entirely in the cytoplasm, the next critical stage of the viral life cycle involves the precise trafficking of disparate viral components to specific subcellular “assembly platforms” for convergence. This complex logistical process is orchestrated entirely by hijacked host transport systems—primarily the cytoskeletal network (e.g., microtubules and actin filaments) and associated vesicular transport systems—to ensure precise spatiotemporal coordination. The selection and establishment of these assembly platforms vary among viruses and can be categorized into two main modes. The first is “internal remodeling”, where the virus establishes a dedicated assembly factory on an internal membrane system. For viruses such as HCV, LLPS mechanism is crucial for coordinating the assembly processes [156]. This creates a highly efficient microenvironment that integrates replication with assembly, allowing nascent viral genomes to be encapsidated and combined with envelope proteins for final virion formation [157]. Furthermore, HCV infection involves hijacking Stress Granule (SG) components, leading to the redistribution of host factors such as G3BP1, ATXN2, DDX3X, and PABP1 to viral replication sites near the lipid droplets [102]. This indicates that LLPS principle controls the precise logistics and molecular aggregation required for encapsidation and final virion formation. In contrast to HCV’s choice of lipid droplets for replication, coronaviruses assemble at the ER-Golgi intermediate compartment (ERGIC) [158]. Here, the viral M protein acts as a central organizer, marshaling the nucleocapsid and membrane proteins and directing budding into the ERGIC lumen to form mature, vesicle-enclosed particles [159]. The second mode is more direct, utilizing the plasma membrane as the final rendezvous point. IAV is a prime example of this strategy. It relies on the host Rab GTPase family (e.g., Rab11a) to guide its vRNPs and envelope proteins to converge at lipid raft microdomains at the apical plasma membrane, in preparation for subsequent budding [160,161,162]. Throughout this process, the fidelity of virion formation is itself directly assisted and precisely regulated by host factors. This regulation spans the entire assembly sequence. It includes “molecular switches” that initiate the process, such as the host kinase SRPK1 and phosphatase PP2A, which control the switch from viral transcription to assembly mode by regulating the phosphorylation of the filovirus VP30 protein [95]. It also involves “molecular glues” that ensure structural integrity; for example, the host-derived small-molecule inositol hexakisphosphate (IP6) and the host protein Cyclophilin A (CypA) are both critical for the proper assembly and subsequent maturation of the HIV capsid [163,164]. Once this virion, meticulously crafted with the aid of host factors, is fully assembled, it is primed to initiate the final step of its egress program: budding from its host cell membrane.

The culmination of the assembly process is the egress of new virions, which must exit the host cell to propagate infection. The mechanism of release is fundamentally dictated by the virus’s structure. Non-enveloped viruses are typically released through a direct and destructive mechanism of cell lysis, whereas enveloped viruses employ a more intricate strategy: budding from a host membrane. This complex process begins with the active transport of the viral core to a designated membrane site, a journey that relies heavily on the hijacking of the host’s own transport systems, such as the cytoskeleton. The nucleocapsids of filoviruses, for instance, even co-opt the host actin polymerization machinery (the Arp2/3 complex) to form “actin comet tails”, which provide rocket-like propulsion for directed transport to the plasma membrane [95]. This transport is highly dependent on the host actin cytoskeleton, with the virus recruiting and activating the Arp2/3 complex and its upstream activators (e.g., WAVE1 and Rac1) to drive the directed movement of the nucleocapsid through the cytoplasm [95].

Following the formation of a viral “bud” at the membrane, the final step of membrane scission reveals diverse solutions evolved by viruses. One widely adopted strategy is the hijacking of the host ESCRT machinery. As the host’s universal “membrane scission tool” [165,166,167], the ESCRT complex is recruited by viruses like HIV-1, via their Gag protein, to the neck of the budding virion to perform the final “cut” [166]. However, many viruses have evolved ESCRT-independent pathways. IAV does not rely on ESCRT, instead utilizing the amphipathic helix of its own M2 protein to actively induce membrane fission [167]. The strategy of coronaviruses is even more distinct; after budding internally at the ER-Golgi intermediate compartment (ERGIC), they fully co-opt the host secretory pathway for final release via exocytosis [159].

Furthermore, the efficiency of the budding process is fine-tuned by the local lipid microenvironment [153,168]. Viruses create a lipid environment more conducive to membrane curvature and scission by hijacking host enzymes such as fatty acid synthase (FASN) or neutral sphingomyelinase 2 (nSMase2). EBOV, for example, depends on phosphatidylserine (PS) on the inner leaflet of the plasma membrane to create a PS-rich microdomain, which serves as an essential “launching pad” for budding [169]. For another critical lipid structure, IAV activates STAT3 to upregulate cholesterol synthesis via SREBP2. These lipids constitute the “Lipid Rafts” on the cell membrane, which provide the necessary physical platform for the assembly and budding of enveloped viruses [170,171,172].

### 5.2. Targeting HDFs in Assembly and Egress

This comprehensive and profound dependence on host cell machinery—from transport and membrane remodeling to lipid metabolism—not only reveals the evolutionary ingenuity of viruses but, more critically, provides a series of strategic entry points for antiviral intervention. Targeting host factors essential for viral budding represents a highly promising strategy for developing broad-spectrum antivirals, wherein drug repurposing to disrupt specific cellular environments required by the virus has shown significant efficacy [173]. In Table 4, we summarize antiviral agents that target key host dependency factors during the viral assembly and egress stages. Drugs that target lipid metabolism and the membrane microenvironment are especially prominent in this area. A prime example is the FDA-approved cardiovascular drug Fendiline, which dismantles the essential “launching pad” for EBOV budding by inhibiting acid sphingomyelinase, thereby disrupting the clustering of phosphatidylserine (PS) at the plasma membrane [169]. Similarly, statins, fatty acid synthase (FASN) inhibitors, and neutral sphingomyelinase 2 (nSMase2) inhibitors—primarily used for treating cancer or metabolic diseases [174,175,176]—are receiving significant attention for their potential to inhibit viral release by altering membrane lipid composition, though their clinical application as antiviral therapies requires further validation. Beyond lipids, viral budding is also dependent on host signaling pathways and secretory systems. For instance, the research tool Brefeldin A, despite its high toxicity precluding clinical use, provides an important proof-of-concept for targeting viruses like coronaviruses that rely on the secretory pathway for release by disrupting the Golgi apparatus [177].

In conclusion, viral assembly and release constitute an interconnected process that is profoundly dependent on the host. Therefore, targeting these critical host factors—such as the IP6 binding site, Rab proteins, nSMase2, or the CRM1 protein—has emerged as one of the most promising and cutting-edge directions for the development of next-generation, broad-spectrum antiviral drugs.

## 6. Conclusions and Outlook

The ongoing evolutionary arms race between virus and host, coupled with the profound reliance of RNA viruses on host cellular machinery, forms the cornerstone for a paradigm shift in antiviral strategy. This review has systematically delineated the mechanisms by which RNA viruses hijack HDFs at each stage of their life cycle—from entry and replication to assembly and egress. Our analysis underscores that targeting these highly conserved host factors presents a compelling alternative to direct-acting antivirals (DAAs), which are inherently vulnerable to viral mutation and drug resistance. The HDF-targeting strategy is distinguished by two cardinal virtues: the potential for broad-spectrum efficacy and a high genetic barrier to resistance. The principal challenge remains the risk of host cytotoxicity, as HDFs are integral to normal cellular physiology. Nevertheless, dissecting the virus–host interactome has unequivocally unveiled a new frontier in our fight against viral diseases, positioning host-targeting therapies as a revolutionary and promising avenue for confronting both current and future viral threats.

Looking ahead, the development of host-targeting antivirals will be guided by the pursuit of precision, safety, and synergy.

**Figure 5:**
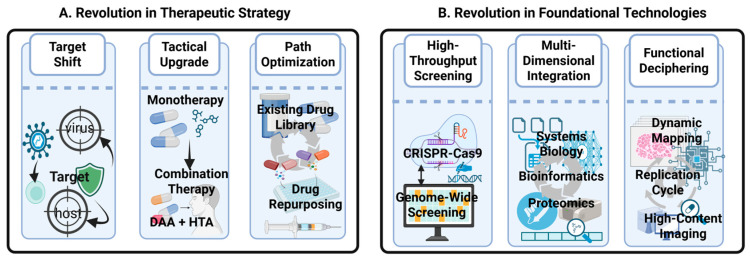
A paradigm shifts in antiviral therapy: new strategies enabled by foundational technologies (Created in BioRender. gh, T. (2025) https://BioRender.com/xr1lnwq) (accessed on 27 November 2025.

1. Synergistic Combination Therapies: The strategic combination of DAAs and HTAs (“DAA + HTA”) will emerge as a cornerstone regimen. This approach constrains viral evolution from multiple angles, potentially allows for lower drug doses, and enhances overall efficacy, representing a robust long-term solution to the problem of drug resistance. Notably, the potential of LLPS targeting is uniquely highlighted by its fundamental orthogonality to the targets of DAAs. The LLPS mechanism is structurally and physicochemical distinct from DAA targeting, making LLPS targeting an excellent candidate for combination therapy (Figure 5A).
2. Tunable Therapeutic Modalities: The field will move beyond simple inhibition towards the “fine-tuned modulation” of HDF activity with regulatory dimensions spanning epigenetics, host signaling pathways, biomolecular condensates and liquid–liquid phase separation, autophagy and autophagy-dependent HDFs, and various host organelles. Modalities such as allosteric modulators and molecular glues aim to precisely perturb the virus–host interface, effectively inhibiting the virus while sparing essential host functions, thereby broadening the therapeutic window.
3. Precision in Target Identification: The next generation of HDFs will be discovered by focusing on those that are “preferentially hijacked” by viruses or become essential only in infected cells. Leveraging functional genomics, proteomics, and AI-driven analytics will be key to identifying these ideal targets with inherently higher therapeutic indices (Figure 5B).

In summary, as our understanding of the dynamic landscape of the virus–host interactome deepens, antiviral therapeutics are entering an unprecedented new era. The precision targeting of host dependency factors across the viral life cycle represents a revolutionary strategy that is poised to provide a powerful scientific arsenal in our ultimate fight against viral diseases.

## Figures and Tables

**Table 1:** The specificity of virus–host recognition, detailing key glycoproteins and receptors used by major RNA viruses for cell entry.

Virus Name	Virus Type	Glycoprotein	Host Receptor
Ebola Virus (EBOV)	−ssRNA	GP	NPC1
Hepatitis C virus (HCV)	+ssRNA	E1/E2	SR-BI, CD81, Claudin-1, Occludin
Severe Acute Respiratory Syndrome Coronavirus 2 (SARS-CoV-2)	+ssRNA	Spike Protein	ACE2
Influenza A Virus (IAV)	−ssRNA	Hemagglutinin, HA	Sialic Acid, mGluR2
Chikungunya Virus (CHIKV)	+ssRNA	E1/E2	MXRA8, Prohibitin-1, TIM-1, Glycosaminoglycans (GAGs)
Sindbis Virus (SINV)	+ssRNA	E1/E2	Laminin Receptor (LAMR), Heparan Sulfate (HS)
Respiratory Syncytial Virus (RSV)	−ssRNA	F, G	CX3CR1, Nucleolin (NCL), Heparan Sulfate (HS)
Norovirus (NoV)	+ssRNA	VP1	Histo-Blood Group Antigens (HBGAs), CD300lf
Enterovirus A71 (EV-A71)	+ssRNA	VP1	SCARB2, PSGL-1, HEV
Coxsackievirus B3 (CVB3)	+ssRNA	VP1	CAR, DAF
Nipah Virus (NiV)	−ssRNA	G, F	Ephrin-B2, Ephrin-B3
Zika Virus (ZIKV)	+ssRNA	E(Envelope)	AXL, Tyro3, TIM-1, DC-SIGN

**Table 2:** Therapeutic strategies targeting key host dependency factors during the viral attachment and entry stage. Among the marketed drugs, there are drugs that have been repurposed.

Host Dependency Factor (HDF)	Virus Example	Clinical States	Targeting Drug/Strategy
ACE2 (Angiotensin-Converting Enzyme 2)	Severe Acute Respiratory Syndrome Coronavirus 2 (SARS-CoV-2)	Marketed drugs In clinical development.	Ivermectin, Paritaprevir, Darunavir, Chloroquine, Hydroxychloroquine, Remdesivir, Azithromycin, Arbidol [67] MLN-4760(in Phase 2 clinical trial) [67], APN01(in Phase 3 clinical trial) [72]
Sialic Acid Receptors	Influenza Virus (IAV)	Marketed drugs In clinical development	Oseltamivir, Zanamivir [73], Peramivir [74], Gemtuzumab, Ozogamicin [75] DAS-181(in Phase 2 clinical trial) [74], NC318 (in Phase 2 clinical trial) [75]
mGluR2 (Metabotropic Glutamate Receptor 2) [44]	Influenza Virus (IAV)	/	/
CCR5	Human Immunodeficiency Virus (HIV)	Marketed drugs	Maraviroc [30]
NPC1 (Niemann-Pick C1)	Ebola Virus (EBOV)	Marketed drugs	Imipramine [76]
SR-BI (Scavenger Receptor Class B Type I)	Hepatitis C Virus (HCV)	In clinical development	ITX5061 (in Phase 2 clinical trial) [77]
CD81	Hepatitis C Virus (HCV)	In clinical development	Civacir (in Phase 3 clinical trial) [78]
Claudin-1 [41]	Hepatitis C Virus (HCV)	/	/
Occludin [41]	Hepatitis C Virus (HCV)	/	/
TMPRSS2 (Transmembrane Serine Protease 2)	Coronaviruses (e.g., SARS-CoV-2)	Marketed drugs	Camostat mesylate, Nafamostat mesylate [79]
Cathepsins	Coronaviruses (e.g., SARS-CoV-2)	Marketed drugs	Clofazimine, Glycopeptide antibiotics, Rifampicin, Saquinavir, Chloroquine, Astaxanthin, Dexamethasone, IFN-γ, Clenbuterol, Heparin [80]
Endosomal Acidification (Low pH)	Various (e.g., Coronaviruses, Influenza Virus)	Marketed drugs	Chloroquine, Hydroxychloroquine [81]
Clathrin [41]	Hepatitis C Virus (HCV)	/	/
Cholesterol Synthesis Pathway	Ebola Virus (EBOV), various	Marketed drugs	Statins [82]
p38 MAPK	Various RNA viruses	/	/
AAK1	SARS-CoV-2	Marketed drugs	Baricitinib [83]
HDAC6	Various RNA viruses	/	/
MXRA8, Prohibitin-1, GAGs	Chikungunya Virus (CHIKV)	/	/
LAMR	Sindbis Virus (SINV)	/	/
CX3CR1, NCL	Respiratory Syncytial Virus (RSV)	/	/
HS [84]	Various (e.g., RSV, SINV)	/	/
HBGAs, CD300lf	Norovirus (NoV)	/	/
SCARB2, PSGL-1, HEV	Enterovirus A71 (EV-A71)	/	/
CAR, DAF	Coxsackievirus B3 (CVB3)	/	/
Ephrin-B2, Ephrin-B3	Nipah Virus (NiV)	/	/
AXL, Tyro3, DC-SIGN	Zika Virus (ZIKV)	/	/
TIM-1	Various (e.g., CHIKV, ZIKV)	/	/
p38 MAPK	Various RNA viruses	/	/

**Table 4:** Therapeutic strategies targeting key host dependency factors during the viral assembly and egress stage. Among the marketed drugs, there are drugs that have been repurposed.

Host Dependency Factor (HDF)	Virus Example	Clinical States	Targeting Drug/Strategy
CRM1	Human Immunodeficiency Virus (HIV), Influenza A virus (IAV)	Marketed drugs	Selinexor/KPT-330 (in Phase 1/2 clinical trial), Verdinexor/KPT-335 (in Phase 1/2 clinical trial) [178]
NXF1 System	Influenza A virus (IAV)	In clinical development	Tapinarof [179]
Rab GTPase Family (e.g., Rab11a) [160,161,162]	Influenza A virus (IAV)	/	/
Inositol Hexaphosphate (IP6) [163,164]	Human Immunodeficiency Virus (HIV)	/	/
Cyclophilin A (CypA)	Human Immunodeficiency Virus (HIV)	In clinical development	Cyclosporin A (CsA) [164]
Host Kinase SRPK1 & Phosphatase PP2A [95]	Filoviruses	/	/
Arp2/3 Complex [95]	Filoviruses	/	/
ESCRT Machinery [165,180,181]	Various RNA viruses	/	/
Fatty Acid Synthase (FASN) [169]	Various (e.g., Ebola Virus (EBOV))	/	/
Neutral Sphingomyelinase 2 (nSMase2) [169]	Various	/	/
Acid Sphingomyelinase (ASM)/PS Clustering	Ebola Virus (EBOV)	In clinical development	Fendiline [169]
Lipid rafts	Influenza Virus (IV)	In clinical development	Gemfibrozil, Lovastatin [182]
G3BP1	HCV	/	/
ATXN2	HCV	/	/
DDX3X	HCV	/	/
PABP1	HCV	/	/

## Data Availability

No new data were created or analyzed in this study. Data sharing is not applicable to this article.
